# Effects of CO_2_ enrichment on benthic primary production and inorganic nitrogen fluxes in two coastal sediments

**DOI:** 10.1038/s41598-017-19051-w

**Published:** 2018-01-18

**Authors:** Kay Vopel, Cintya Del-Río, Conrad A. Pilditch

**Affiliations:** 1 School of Science, Auckland University of Technology, Private Bag, 92006 Auckland, New Zealand; 2 School of Science, University of Waikato, Private Bag, 3105 Hamilton, New Zealand

## Abstract

Ocean acidification may alter the cycling of nitrogen in coastal sediment and so the sediment–seawater nitrogen flux, an important driver of pelagic productivity. To investigate how this perturbation affects the fluxes of NO_X_^−^ (nitrite/nitrate), NH_4_^+^ and O_2_, we incubated estuarine sand and subtidal silt in recirculating seawater with a CO_2_-adjusted pH of 8.1 and 7.9. During a 41-day incubation, the seawater kept at pH 8.1 lost 97% of its NO_X_^−^ content but the seawater kept at pH 7.9 lost only 18%. Excess CO_2_ increased benthic photosynthesis. In the silt, this was accompanied by a reversal of the initial NO_X_^−^ efflux into influx. The estuarine sand sustained its initial NO_X_^−^ influx but, by the end of the incubation, released more NH_4_^+^ at pH 7.9 than at pH 8.1. We hypothesise that these effects share a common cause; excess CO_2_ increased the growth of benthic microalgae and so nutrient competition with ammonia oxidising bacteria (AOB). In the silt, diatoms likely outcompeted AOB for NH_4_^+^ and photosynthesis increased the dark/light fluctuations in the pore water oxygenation inhibiting nitrification and coupled nitrification/denitrification. If this is correct, then excess CO_2_ may lead to retention of inorganic nitrogen adding to the pressures of increasing coastal eutrophication.

## Introduction

Ocean acidification, a consequence of the absorption of atmospheric carbon dioxide, is expected to accelerate over the upcoming century, altering marine biota and associated ecosystem processes^1–3^. Our ability to predict the functioning of the future high-CO_2_ ocean, however, is still in its infancy^1,4,5^. For the coastal ocean, such prediction is complicated by natural and anthropogenic phenomena rendering the seawater carbonate chemistry variable at timescales from seconds to years (reviewed by Waldbusser & Salisbury^6^, Mostofa *et al*.^3^). Current research thus distinguishes between carbonate weather—the short-term variability in the seawater pH–*p*CO_2_ system—and carbonate climate, the longer-term shift in the baseline pH–*p*CO_2_ system^6^. The latter may alter environmental conditions for microorganisms in coastal sediment that drive the remineralisation of organic matter and associated biogeochemical cycles^7^. Given that benthic remineralisation provides 30–80% of the inorganic nutrients required by pelagic primary production^8–10^, ocean acidification-driven alterations of benthic nutrient cycles could have implications for coastal ecosystem functioning. The nitrogen cycle may be altered more than any other nutrient cycle in response to CO_2_ enrichment (reviewed by Hutchins *et al*.^5^). For example, studies have revealed evidence for CO_2_-induced changes in the sediment–seawater inorganic nitrogen flux^11,12^, increased nitrogen fixation due to enhanced growth of diazotrophic cyanobacteria^13–15^, and inhibition of nitrification^7^. Other studies, however, found no effects of CO_2_ enrichment on benthic nitrification^16^ and denitrification^17^.

Unravelling the mechanisms behind CO_2_-induced changes in benthic nitrogen cycling is challenging because the microbial activity that drives nitrogen transformations is modulated by macrobenthic infauna reworking particles and ventilating burrows^11,18–26^. In coastal waters where sunlight penetrates to the seabed, another complication exists: excess CO_2_ may increase the photosynthesis of benthic microalgae^27,28^ altering the activity of nitrogen transforming microbes and associated sediment–seawater inorganic nitrogen flux. Two mechanisms have been proposed; (1) benthic microalgae may outcompete ammonia-oxidizing microorganisms in the uptake of ammonia^29,30^, and (2) enhanced benthic O_2_ evolution may inhibit dissimilatory nitrate reduction^29–33^. Although the effects of CO_2_ enrichment on benthic microalgae has received some attention^27,34,35^ the subsequent effects on inorganic nitrogen fluxes are unknown.

**Figure 1:**
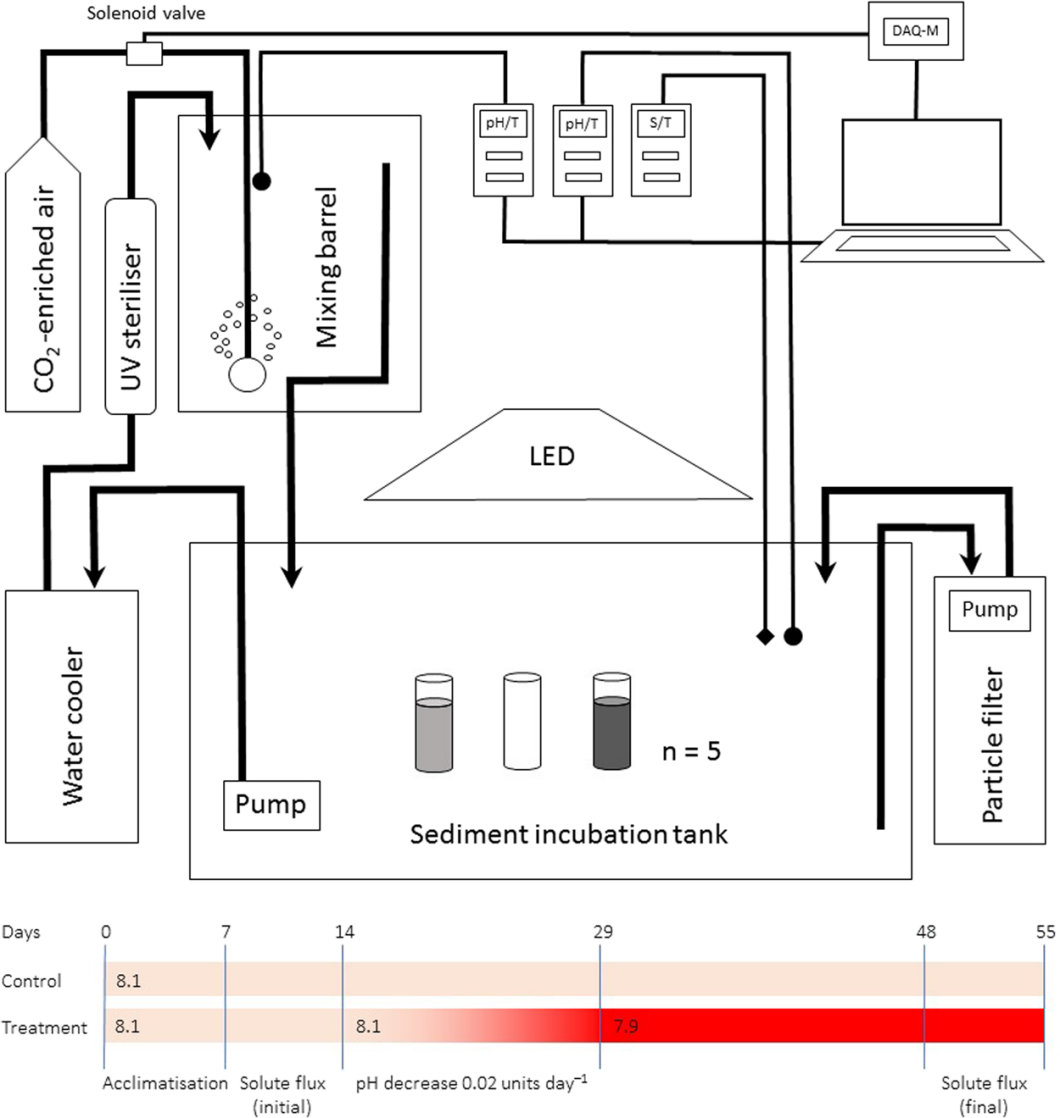
Experimental setup and timeline. Upper panel: Diagram showing one of two identical temperature-pH controlled experimental units that held the sediment cores. Software kept the pH of the seawater in the mixing barrel at a setpoint by opening and closing a solenoid valve that controlled the addition of CO_2_-enriched air. Overhead LED’s illuminated cores on a 12 h light-dark cycle and water circulated via pumps ensured units were well mixed. Lower panel: Timeline of the experimental procedures. Numbers in bars indicate the pH of the seawater in the mixing barrel.

Here, we report the results of a laboratory experiment in which we incubated intact field-collected cores of two contrasting but common coastal sediments; an estuarine sand and a subtidal silt (Table S1), to investigate how CO_2_ perturbation affects the sediment–seawater O_2_ and inorganic nitrogen fluxes. These sediment types span the range observed coastally and differ substantially in their biogeochemistry, microalgae biomass and production as well as inorganic nitrogen fluxes^36–38^. Because of these differences, we expected them to respond differentially to CO_2_ enrichment. We submerged cores of each sediment type into two seawater-circulating experimental units (hereafter, Control and Treatment, Fig. 1) for a total experimental period of 55 d. Initially the pH of the seawater in both units was set to 8.1 and after a 7-d acclimatisation period, we measured the sediment–seawater exchange of O_2_, ammonium (NH_4_^+^), and nitrite/nitrate (NO_X_^−^) under conditions of light and darkness (hereafter, *initial*). On day 15, we lowered the pH of the seawater in the Treatment by 0.02 units per day until a pH of 7.9 was reached, a pH predicted for the end of century^39^. This pH was maintained until completion of the experiment. During the last week of the experiment, we repeated the initial solute flux measurements (hereafter, *final*).

Our measurements revealed evidence for CO_2_-induced increase in benthic photosynthesis and a reversal of the sediment–seawater flux of NO_X_^−^. To explain these results, we propose that CO_2_-enhanced photosynthesis of benthic microphytes inhibits nitrification and coupled nitrification/denitrification. If this mechanism is correct, then CO_2_ enrichment of coastal seawater may lead to the retention of nitrogen, adding to the pressures of increasing coastal eutrophication.

## Results

### Seawater inorganic nitrogen

We expected the inorganic nitrogen content of the seawater circulating in the both Treatment and Control experimental units to change over time due to the sediment’s metabolic activity and gas exchange with the atmosphere. Water column measurements confirmed such change and revealed that, at the end of the experiment, Control seawater had lost more inorganic nitrogen than the pH-reduced Treatment seawater (Table 1). In the Control experimental unit, the *final* [NH_4_^+^] and [NO_X_^−^] were respectively 13 and <3% of the *initial* concentration and significantly lower (*p*_perm_ < 0.0001) than the final concentrations in the Treatment seawater, which were 42 and 82% of the initial concentrations, respectively.

**Table 1:** Seawater properties. Initial and final average (±1 s.d.) seawater pH_T_, total alkalinity (A_T_), dissolved inorganic carbon (DIC), partial CO_2_ pressure (pCO_2_), ammonium (NH_4_^+^) and nitrate/nitrite (NO_X_^−^) concentrations in Control and Treatment seawater. Number of replicate measurements are given in parentheses except for pH_T_ data where n is >4 × 10^5^ and 3 × 10^4^ for initial and final readings respectively.

	Control		Treatment	
	Initial	Final	Initial	Final
pCO_2_ (μatm)	540 ± 66 (4)	632 ± 40 (5)	558 ± 55 (4)	1324 ± 76 (5)
pH_T_	8.10 ± 0.02	8.10 ± 0.02	8.10 ± 0.02	7.87 ± 0.03
DIC (mmol kg^−1^)	3.91 ± 0.03 (4)	3.87 ± 0.09 (5)	3.91 ± 0.04 (4)	4.16 ± 0.03 (5)
A_T_ (mmol kg^−1^)	4.38 ± 0.03 (4)	4.33 ± 0.09 (5)	4.38 ± 0.04 (4)	4.44 ± 0.04 (5)
NH_4_^+^ (mg m^−3^)	132 ± 29 (8)	17.7 ± 9.7 (12)	137 ± 27 (8)	57 ± 27 (11)
NO_x_^−^ (mg m^−3^)	188 ± 7 (8)	5.0 ± 3.3 (12)	189 ± 16 (8)	156 ± 2.7 (11)

### Total sediment O_2_ uptake and inorganic nitrogen flux

#### Estuarine sand

**Figure 2:**
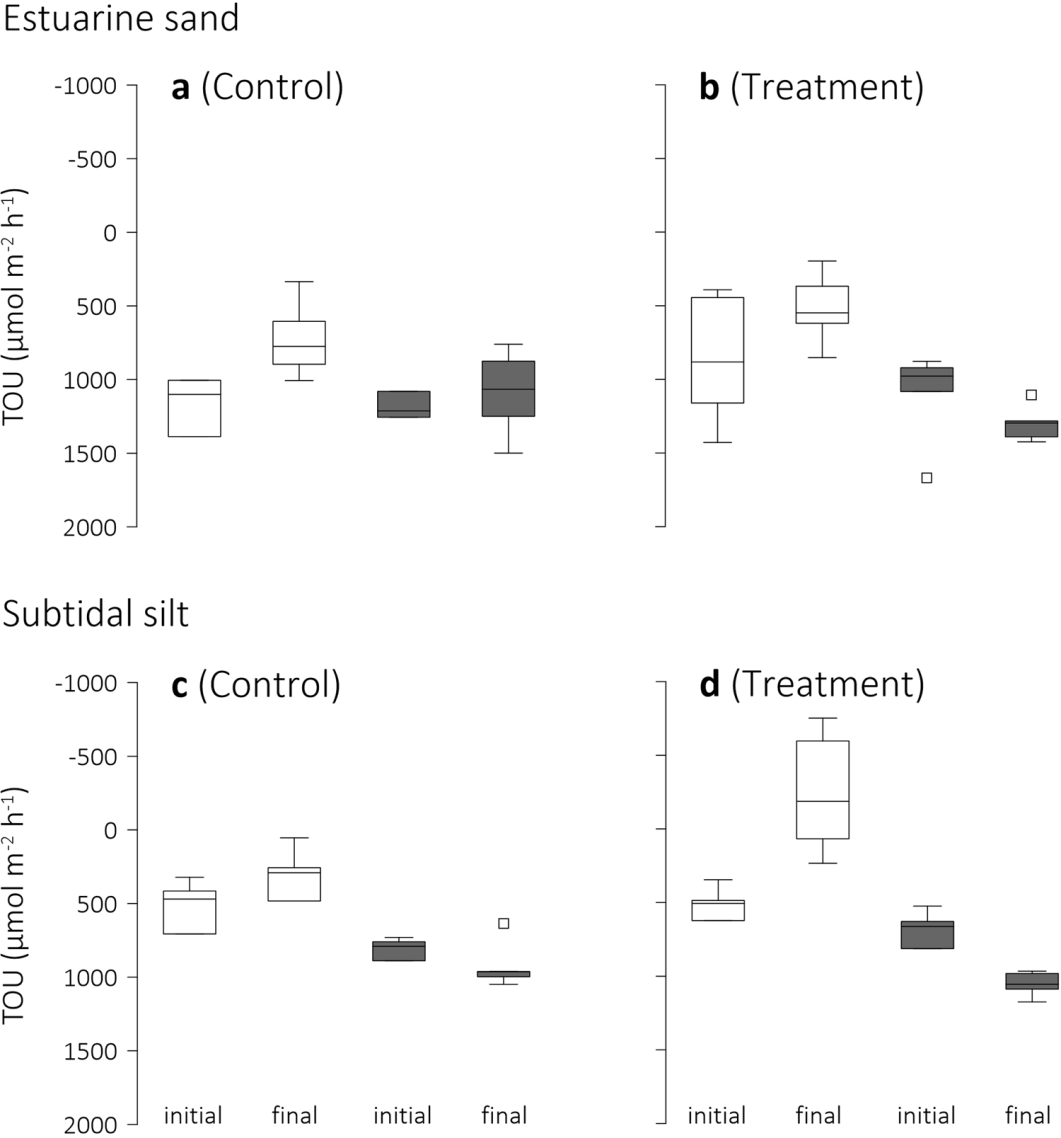
Total sediment O_2_ uptake. Box-Whisker plots of the initial and final total O_2_ uptake (TOU) of (**a**,**b**) estuarine sand and (**c**,**d**) subtidal silt under conditions of darkness (grey boxes) and light in Control and Treatment seawater. The initial seawater pH was 8.1 whereas the final seawater pH was 8.1 in Control and 7.8 in Treatment. Note negative values indicate a sediment efflux of O_2_, positive values an influx. Whiskers indicate minimum and maximum TOU and square symbols, outliers.

The sand’s *initial* total oxygen uptake, TOU, was similar under conditions of light and darkness and similar in Treatment and Control experimental units (Fig. 2a,b, Table 2a). (Note that throughout, a negative flux is defined as a removal of a solute from the sediment and conversely a positive flux indicates uptake, that is movement of solutes from the seawater into the sediment). Because TOU_light_ did not differ from TOU_dark_, this sand initially classified as fully heterotrophic (Benthic Trophic State Index^40^, BTSI = 0). During the experiment, however, TOU_light_ had decreased in both Control and Treatment, while TOU_dark_ remained unchanged (Fig. 2a,b, Table 2b). Consequently, at the completion of the experiment, this sand classified as net heterotrophic (BTSI = 1, 0 < TOU_light_ < TOU_dark_). The conditions under which this sand was incubated—reduced physical disturbance following the isolation of the sand from its intertidal environment, and elevated dissolved inorganic carbon (DIC, Table 1) compared to the DIC at the sampling site (2015 annual average^41^ = 2.1 μmol kg^−1^)—appeared to enhance benthic photosynthesis in both, Control and Treatment experimental units. The difference between TOU_dark_ and TOU_light_ (∆TOU = TOU_dark_ − TOU_light_), a proxy of the contribution of benthic microalgae to the overall O_2_ flux, was larger in Treatment than in Control, and larger at the completion of the experiment than at the start, but no interaction between time and pH treatment was detected (Table 2b). Separate analyses of fluxes in Control and Treatment seawater (Table 2c), however, revealed that while in Control seawater TOU_light_ and TOU_dark_ did not differ throughout the experiment, in Treatment seawater, TOU_light_ and TOU_dark_ differed significantly at completion of the experiment. This indicates that the CO_2_-enriched Treatment seawater (Table 1) increased benthic photosynthesis more than the Control seawater.

**Table 2:** Summary statistics. Summary of repeated measures two-way PERMANOVA’s testing for the effects of pH treatment (Control (CTR), Treatment (TRE)), time (initial (INI), final (FIN)) and light regime (light (LIG), dark (DAR)) on sediment total O_2_ uptake (TOU), light/dark differences in TOU (∆TOU), and the flux of ammonium (NH_4_^+^) and nitrate/nitrite (NO_X_^−^). For each sediment type (estuarine sand (ES) or subtidal silt (SS)) the effects of two factors on a response variable were tested within the different levels of the third factor. The first column of the table indicates the response variable-sediment type-third factor combination followed by the p_perm_ values for the two tested factors. Results of post-hoc tests (significant interaction term) (p_perm_ < 0.05) are given where appropriate. NS = not significant (p_perm_ > 0.2).

Response					Pair-wise or Post-hoc test results
**(a) pH treatment and light regime within time**					
	**pH**	**Light**	**Core(pH)**	**pH** × **Light**	
TOU-ES-INI	NS	NS	NS	NS	
TOU-ES-FIN	NS	<0.001	0.0011	<0.0012	LIG(CTR = TRE), DAR(CTR = TRE); CTR(DAR > LIG), TRE(DAR > LIG)
NH_4_^+^-ES-INI	NS	NS	0.011	NS	
NH_4_^+^-ES-FIN	0.0394	0.0118	NS	NS	TRE < CTR, DAR < LIG
NO_x_^−^-ES-INI	NS	NS	NS	NS	
NO_x_^−^-ES-FIN	0.0081	NS	0.0017	NS	CTR < TRE
TOU-SS-INI	NS	0.0104	NS	NS	DAR > LIG
TOU-SS-FIN	0.103	<0.001	NS	0.019	LIG(CTR > TRE), DAR(CTR = TRE); CTR(DAR > LIG), TRE(DAR > LIG)
NH_4_^+^-SS-INI	NS	NS	NS	NS	
NH_4_^+^-SS-FIN	0.015	0.0493	NS	NS	CTR < TRE, DAR < LIG
NO_x_^−^-SS-INI	NS	0.0014	NS	NS	DAR < LIG
NO_x_^−^-SS-FIN	0.0083	0.045	NS	NS	CTR < TRE; DAR < LIG
**(b) pH treatment and time within light regime**					
	**pH**	**Time**	**Core(pH)**	**pH** × **Time**	
TOU-ES-DAR	NS	NS	NS	NS	
TOU-ES-LIG	NS	0.006	NS	NS	FIN < INI
∆TOU-ES	0.016	0.0073	NS	NS	CTR < TRE, INI < FIN
NH_4_^+^-ES-DAR	NS	NS	NS	NS	
NH_4_^+^-ES-LIG	NS	NS	NS	NS	
NO_x_^−^-ES-DAR	0.0287	0.0031	NS	0.0155	INI(CTR = TRE), FIN(CTR < TRE); CTR(FIN < INI), TRE(INI = FIN)
NO_x_^−^-ES-LIG	0.0069	0.0095	NS	NS	CTR < TRE; FIN < INI
TOU-SS-DAR	NS	0.0099	NS	0.059	FIN > INI
TOU-SS-LIG	NS	0.0053	NS	0.047	CTR(INI = FIN), TRE(INI = FIN); INI(CTR = TRE), FIN(TRE < CTR)
∆TOU-SS	NS	0.0022	NS	0.019	INI(CTR = TRE), FIN(CTR < TRE); CTR(INI = FIN), TRE(INI < FIN)
NH_4_^+^-SS-DAR	NS	NS	NS	NS	
NH_4_^+^-SS-LIG	NS	NS	NS	NS	
NO_x_^−^-SS-DAR	0.0415	0.003	NS	0.0023	INI(CTR = TRE), FIN(CTR < TRE); CTR(FIN = INI), TRE(INI < FIN)
NO_x_^−^-SS-LIG	0.0258	0.0015	NS	0.0059	INI(CTR = TRE), FIN(CTR < TRE); CTR(FIN = INI), TRE(INI < FIN)
**(c) Light regime and Time within pH treatment**					
	**Light**	**Time**	**Core(Light)**	**Light** × **Time**	
TOU-ES-CTR	NS	NS	NS	NS	
TOU-ES-TRE	0.031	NS	NS	0.026	DAR(INI = FIN), LIG(INI = FIN); INI(DAR = LIG), FIN(DAR > LIG)
NH_4_^+^-ES-CTR	NS	NS	NS	NS	
NH_4_^+^-ES-TRE	NS	NS	NS	NS	
NO_x_^−^-ES-CTR	NS	<0.001	NS	NS	FIN < INI
NO_x_^−^-ES-TRE	NS	NS	NS	NS	
TOU-SS-CTR	0.0094	NS	NS	NS	
TOU-SS-TRE	0.003	NS	NS	0.003	DAR(INI < FIN), LIG(INI = FIN); INI(DAR = LIG), FIN(DAR > LIG)
NH_4_^+^-SS-CTR	NS	NS	NS	NS	
NH_4_^+^-SS-TRE	0.0289	NS	NS	NS	DAR < LIG
NO_x_^−^-SS-CTR	0.0075	0.047	NS	NS	DAR < LIG; INI < FIN
NO_x_^−^-SS-TRE	0.009	<0.001	NS	0.0376	DAR(INI < FIN), LIG(INI < FIN); INI(DAR < LIG), FIN(DAR = LIG)

**Figure 3:**
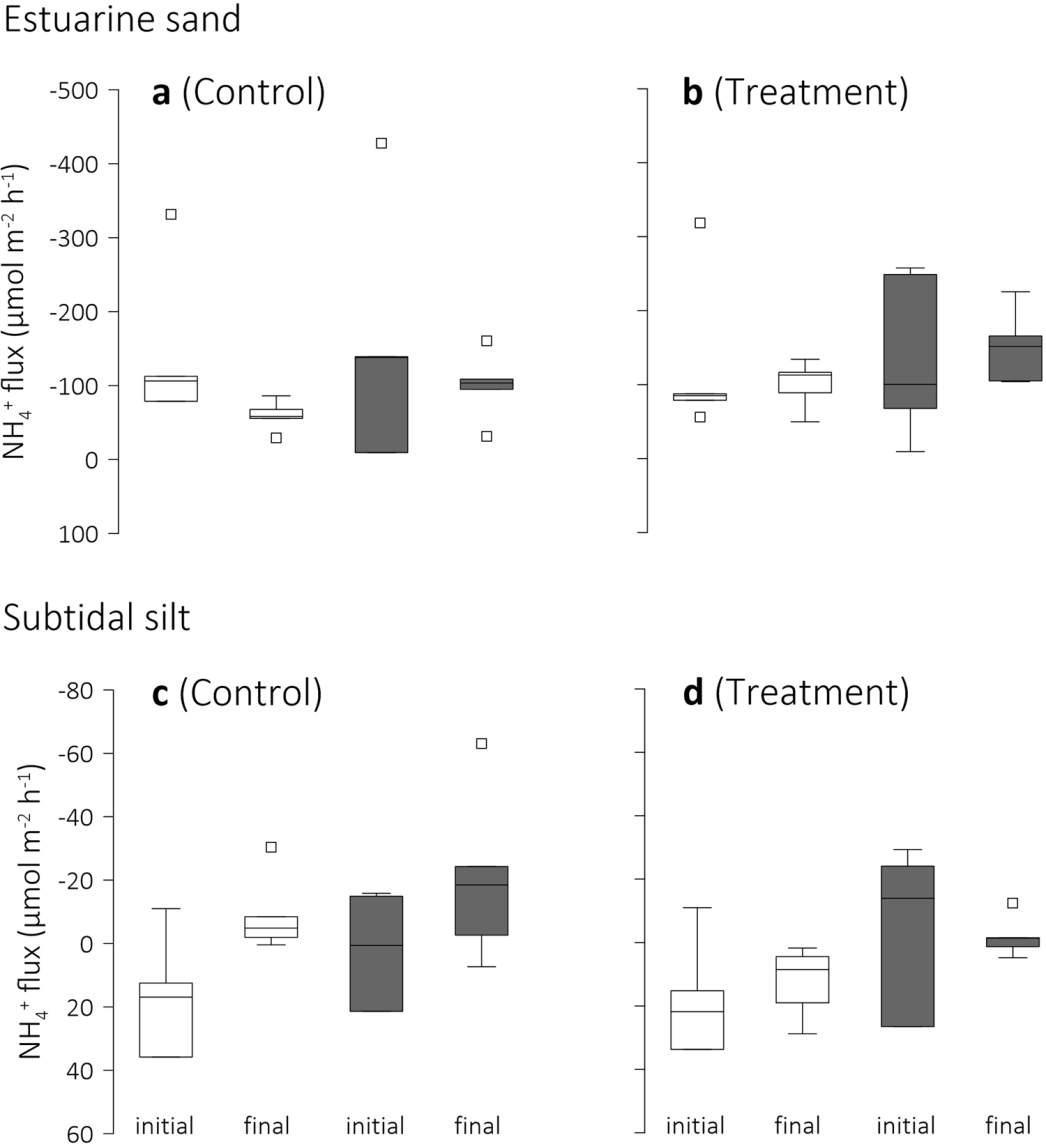
Sediment–seawater flux of ammonium. Box-Whisker plots of the initial and final sediment NH_4_^+^ uptake (positive values, influx) or release (negative values efflux) of (**a**,**b**) estuarine sand and (**c**,**d**) subtidal silt under conditions of darkness (grey boxes) and light in Control and Treatment seawater. The initial seawater pH was 8.1 whereas the final seawater pH was 8.1 in Control and 7.8 in Treatment. Note differences in scale between plots. Whiskers indicate minimum and maximum flux and square symbols indicate outliers.

The evidence for enhanced benthic photosynthesis in Control and Treatment seawater was supported by our NH_4_^+^ flux measurements. The *initial* release of NH_4_^+^ from the estuarine sand (negative fluxes in Fig. 3) was similar in Control and Treatment, and similar under conditions of light and darkness (Fig. 3a,b, Table 2a). At the completion of the experiment, however, the sand released significantly more NH_4_^+^ in darkness than in light (Fig. 3a,b, Table 2a), indicating that the influence of benthic algae on this flux had increased.

**Figure 4:**
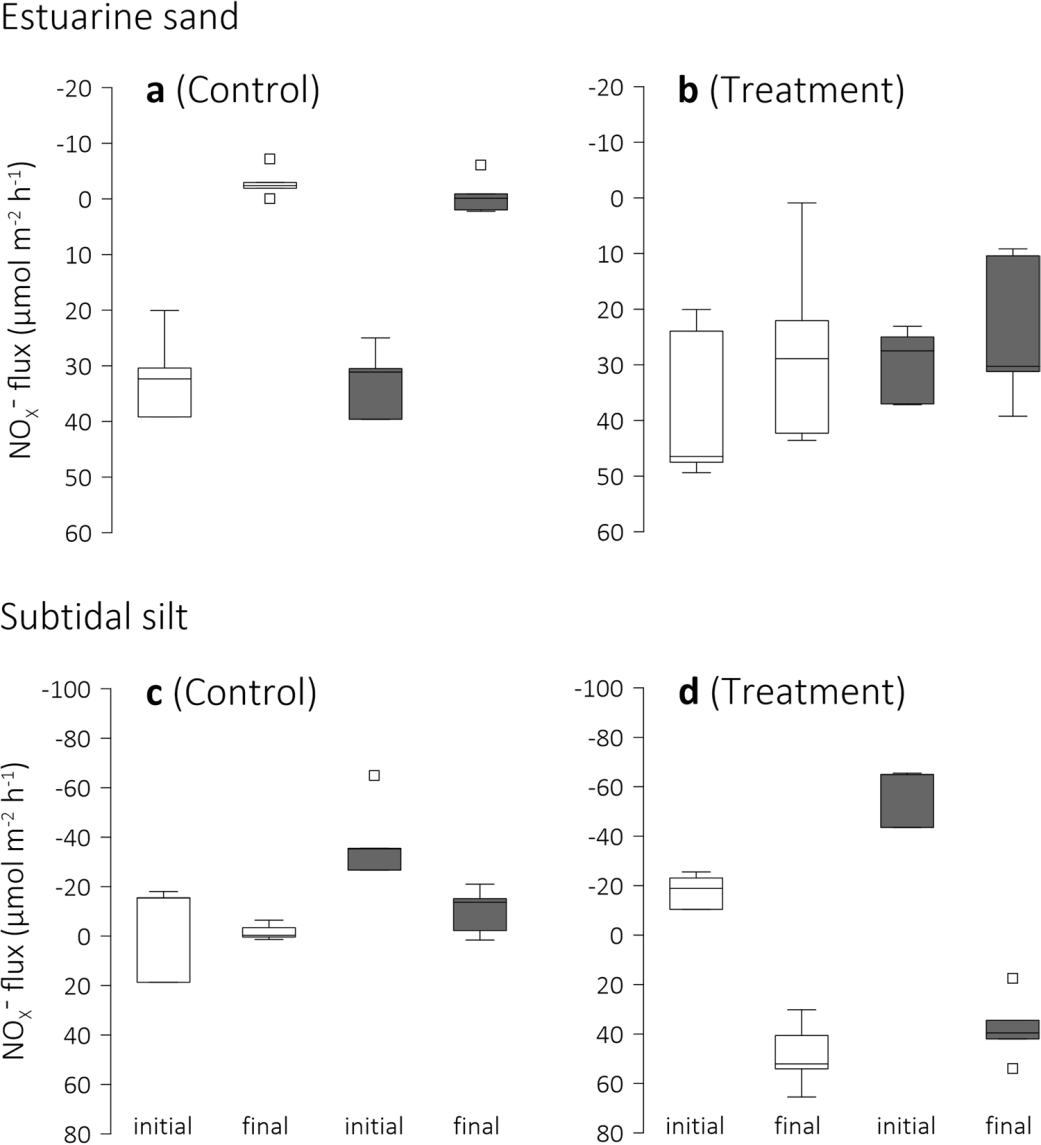
Sediment–seawater flux of nitrite/nitrate. Box-Whisker plots of the initial and final sediment NO_X_^−^ uptake (positive values, influx) or release (negative values, efflux) of (**a**,**b**) estuarine sand and (**c**,**d**) subtidal silt under conditions of darkness (grey boxes) and light in Control and Treatment seawater. The initial seawater pH was 8.1 whereas the final seawater pH was 8.1 in Control and 7.8 in Treatment. Note differences in scale between plots. Whiskers indicate minimum and maximum flux and square symbols indicate outliers.

A significant Control–Treatment difference was also observed for the *final* NO_X_^−^ fluxes (Table 2a). Our *initial* flux measurements again confirmed that the sand’s NO_X_^−^ flux was similar in Control and Treatment and similar under conditions of light and darkness (Fig. 4a,b, Table 2a). At completion of the experiment, the NO_X_^−^ flux was still independent of light regime, however, in Control it had reversed from uptake to small release (Fig. 4a). In Treatment, this flux remained unchanged (Fig. 4b, Table 2a,c).

Overall, our solute flux data revealed that the experimental conditions increased photosynthesis at the surface of the estuarine sand submerged in both Control and Treatment seawater. The conditions altered the fluxes of NH_4_^+^ in Treatment seawater and NO_X_^−^ in Control seawater to an extent that, at the completion of the experiment, they differed significantly from the fluxes measured in Control and Treatment, respectively. Furthermore, there is evidence that the CO_2_-enriched Treatment seawater enhanced benthic photosynthesis more that the Control seawater.

#### Subtidal silt

Changes in the silt’s TOU suggest a positive effect of CO_2_ enrichment on benthic photosynthesis. In contrast to the estuarine sand, the silt’s *initial* TOU_dark_ exceeded TOU_light_ in both, Control and Treatment seawater, indicating that benthic microalgae contributed to the silt–seawater O_2_ flux (Fig. 2c,d, Table 2a). TOU in Control and Treatment seawater were similar and, because 0 < TOU_light_ < TOU_dark_, this silt classified as net heterotrophic (BTSI = 1). During the experiment, the silt in Control seawater remained net heterotrophic, while the silt in Treatment seawater became net autotrophic (BTSI = 2, TOU_light_ < 0 and |TOU_light_| < TOU_dark_) and, on average, released O_2_ into the overlying seawater (Fig. 2d). This difference in trophic state occurred because the silt’s TOU_dark_ had increased, in both Control and Treatment (Table 2b), and the final TOU_light_ was lower in Treatment than in Control (Table 2a). Consequently, at the completion of the experiment, ∆TOU was significantly larger in Treatment than in Control (Fig. 2c,d, Table 2b).

**Figure 5:**
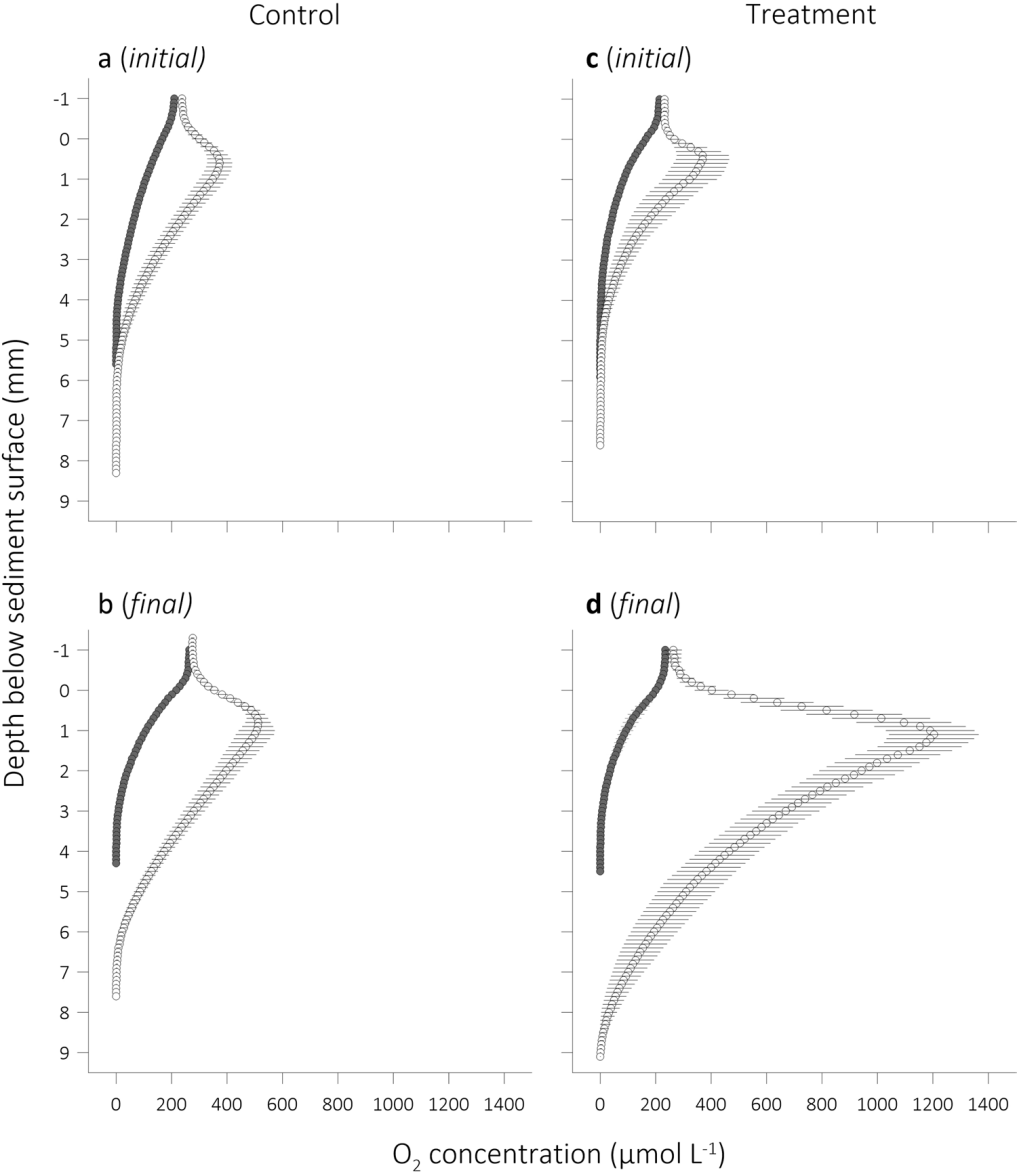
Sediment pore water oxygenation. Average ± 1 s.d. (n = 10) vertical [O_2_] microprofiles measured in a single subtidal silt core submerged in either Control (**a**,**b**) or Treatment (**c**,**d**) seawater, at the beginning (initial; **a**,**c**) and the end (final; **b**,**d**) of the experiment. The measurements were performed under conditions of light (white circles) and darkness (grey circles).

Our microprofiling measurements of the silt’s pore water [O_2_] supported the evidence for a positive effect of excess CO_2_ on benthic photosynthesis (Fig. 5). The microprofiles revealed that benthic photosynthesis during light conditions supersaturated the pore water of the upper silt with O_2_ and, *initially*, increased the penetration of O_2_ by ~2 mm (compare OPD_dark_ and OPD_light_ in Table 3, Fig. 5a,c). The resulting O_2_ efflux from the silt was not sufficient to offset the total silt O_2_ uptake (which includes fauna mediated O_2_ uptake) in either Control and Treatment seawater resulting in a positive TOU_light_ value (i.e. overall O_2_ uptake, Fig. 2c,d). By the end of the experiment, the subsurface [O_2_] peak had increased in both Control and Treatment seawater, but this increase was much more pronounced in Treatment seawater (3.3× cf 1.4×, Fig. 5b,d). Consistent with the enhanced photosynthesis, there was a marked increase in the difference between the light and dark O_2_ penetration depth from 1.6 to 5.2 mm, but in Control seawater this difference increased from 2.4 mm to only 3.2 mm (Table 3). The increased efflux of O_2_ in Treatment cores must have offset the total silt O_2_ uptake resulting in negative TOU_light_ (i.e. overall O_2_ release, Fig. 2d) and altered the trophic status of the silt.

**Table 3:** Sediment total O_2_ uptake and O_2_ penetration depth. Average ± 1 s.d. (n) Initial and final light/dark differences in the total O_2_ uptake (∆TOU = TOU_dark_ − TOU_light_, µmol m^−2^ h^−1^) of estuarine sand and subtidal silt in Control and Treatment seawater. Pore water oxygenation (O_2_ penetration depth, OPD, mm) in light and dark are given for the subtidal silt in Control and Treatment seawater. Number of replicate measurements are given in parentheses and note that the OPD was estimated from repeated (initial, final) measurements of replicate O_2_ microprofiles in one Control and one Treatment core.

	Estuarine sand				Subtidal silt			
	Control		Treatment		Control		Treatment	
	Initial	Final	Initial	Final	Initial	Final	Initial	Final
∆TOU	−46 ± 265 (4)	351 ± 100 (5)	245 ± 435 (5)	845 ± 82 (5)	308 ± 187 (4)	763 ± 98 (5)	171 ± 174 (5)	1438 ± 318 (5)
OPD_light_	—	—	—	—	7.6 ± 0.7 (10)	7.2 ± 0.3 (10)	6.3 ± 0.9 (10)	9.4 ± 0.4 (10)
OPD_dark_	—	—	—	—	5.2 ± 0.3 (10)	4.0 ± 0.3 (10)	4.7 ± 0.6 (10)	4.2 ± 0.3 (10)

As observed for the estuarine sand, the *initial* NH_4_^+^ fluxes of the silt in Control and Treatment were similar and between-core variability rendered the expected differences between light and dark flux insignificant (Fig. 3c,d, Table 2a). Note, that the silt’s NH_4_^+^ fluxes were an order of magnitude smaller than the fluxes of the estuarine sand. Although lacking statistical support, under conditions of light, the silt on average removed NH_4_^+^ from the overlying seawater (there was one exception in each, Control and Treatment), whereas in darkness, both small uptake and release were recorded. At completion of the experiment, the between-core variability had decreased and the NH_4_^+^ uptake in Treatment was significantly larger in light than in darkness (Fig. 3d, Table 2c), indirectly supporting the evidence for a positive effect of CO_2_ enrichment on benthic photosynthesis. Furthermore, at the end of the experiment, the silt’s NH_4_^+^ flux in Treatment seawater was significantly larger than that in Control seawater (Table 2a). That is, CO_2_ enrichment of the seawater sustained Treatment cores as a sink of NH_4_^+^ (and induced larger diel fluctuations), whereas the conditions in Control on average reversed this flux so the cores were a source of NH_4_^+^.

This trend of sustained and changed inorganic nitrogen fluxes in Treatment and Control seawater, respectively, was not apparent in the subtidal silt’s NO_X_^−^ flux. Whereas the estuarine sand *initially* served as sink for seawater NO_X_^−^, the silt provided a source, releasing significantly more NO_X_^−^ under conditions of darkness than under conditions of light (Fig. 4c,d, Table 2a). By the end of the experiment, however, the flux in Treatment had reversed, so that the silt became a sink for NO_X_^−^ (Fig. 4d). In contrast, the silt in Control kept releasing NO_X_^−^ although the flux had decreased significantly (Table 2a,c).

## Discussion

**Figure 6:**
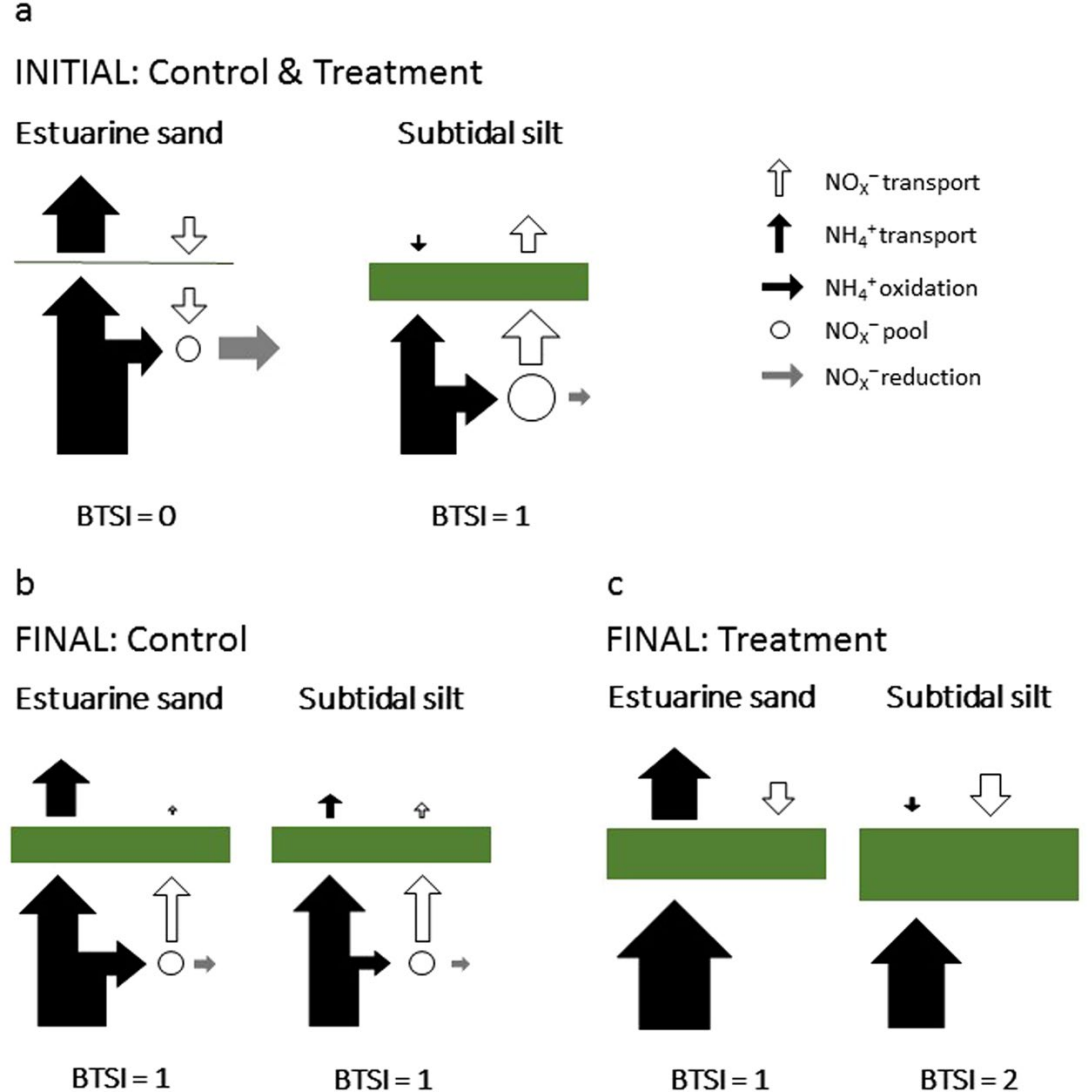
Simplified conceptual model explaining variations in sediment–seawater flux of NO_X_^−^ and NH_4_^+^. The thickness of the green bars indicates the relative influence of microalgae on the sediment–seawater nitrogen flux. The white (NO_X_^−^) and black (NH_4_^+^) arrows above the green bars represent the relative magnitude and direction of the measured fluxes. Vertical arrows beneath the green bars indicate the inferred magnitude and direction of the NO_X_^−^ (white) and NH_4_^+^ (black) transport. Horizontal bars indicate nitrogen oxidation and reduction processes that contribute to the pool of pore water NO_X_^−^ (circle). BTSI, benthic trophic state index.

Our solute flux measurements revealed a differential response of the two sediments to CO_2_ perturbation. At completion of our experiment, excess CO_2_ in the Treatment seawater had enhanced photosynthesis at the surface of both sediment types. This effect was most pronounced, however, in the algae-dominated silt and was accompanied by a reversal of the NO_x_^−^ efflux (Fig. 6). In contrast, the estuarine sand sustained its initial NO_X_^−^ uptake but released more NH_4_^+^ in Treatment seawater than in Control seawater. We hypothesise that these effects shared a common cause; excess CO_2_ enhanced the growth of benthic microalgae. In the silt, the growing assemblage of motile pennate diatoms outcompeted ammonia oxidising bacteria (AOB) for NH_4_^+^ and increased the dark/light fluctuations in the pore water oxygenation inhibiting nitrification. Consequently, the pore water [NO_x_^−^] decreased reversing the initial NO_x_^−^ efflux. Inhibition of nitrification may also explain why, at completion of the experiment, the estuarine sand released more NH_4_^+^ in Treatment seawater than in Control seawater (Table 2a), despite a much higher seawater [NH_4_^+^] in the Treatment experimental unit (~3 × Control seawater [NH_4_^+^], Table 1).

The following discussion relies on the assumption that for both sediment types solute exchange with the sediment-overlying seawater was mainly by molecular diffusion. This assumption is appropriate as the estuarine sand is poorly sorted with a substantial (8%) silt/clay content (Table S1), which would clog pore spaces reducing permeability (e.g., Bartzke *et al*.^42^, Staudt *et al*.^43^). Furthermore, previous pore water profiling measurements in the estuarine sand^44^ and the measurements shown in Fig. 5 did not reveal evidence of significant pore water advection. Below we first discuss the initial sediment–seawater nitrogen fluxes in context of the sediment’s trophic states. We then argue that the changes in fluxes observed in Control seawater follow the depletion of the seawater inorganic nitrogen content. Finally, we discuss the proposed causal relationship between CO_2_ enrichment of the Treatment seawater, enhanced benthic photosynthesis, and the observed changes in the sediment–seawater exchange of inorganic nitrogen.

### Initially complementary nitrogen fluxes coupled sand and silt

Our initial flux measurements revealed that estuarine sand and subtidal silt were coupled by complementary nitrogen fluxes. In both experimental units, the [NH_4_^+^] in the pore water of the estuarine sand must have exceeded that in the overlying seawater so that this sand released NH_4_^+^ (Figs 3a,b and 6a). The [NH_4_^+^] in surface layers of the subtidal silt, however, must have been low resulting in a small uptake from the overlying seawater (Figs 3c,d and 6a). The subtidal silt contained more organic matter than the estuarine sand (Table 1), so one would expect high pore water [NH_4_^+^] in the deeper layers and a greater NH_4_^+^ efflux, but it appears that surface microalgae intercepted (assimilated) the upwardly diffusing NH_4_^+^. In addition, their O_2_ production increased the depth of the oxic/anoxic boundary and so the diffusion distance for NH_4_^+^ from the anoxic layer into the overlying seawater lowering the availability of NH_4_^+^ in the photic zone.

Inspection of surface sediment revealed that the silt was dominated by motile pennate diatom species of the genera *Pleurosigma*, *Gyrosigma*, *Nitzschia*, *Thalassionema*, and *Bacillaria* (M. A. Harper, pers. comm.). The NH_4_^+^ demand of these diatoms may have initially been satisfied by supply from the overlying seawater and the silt’s pore water, reducing competition with AOB for NH_4_^+^ diffusing upwards from the deeper pore water^30^. Under such conditions, diatom-induced oxygenation of the pore water may have stimulated nitrification^33,45–47^ resulting in NO_X_^−^ efflux from the silt (Fig. 6a). This initial loss of NO_X_^−^ from the silt suggests a minor contribution of anaerobic reduction pathways (i.e. denitrification and dissimilatory nitrate reduction to ammonium, DNRA) in removing NO_X_^−^ from the pore water. The diatoms may have also intercepted some of the NO_X_^−^ efflux^33^, as indicated by the significant light/dark differences in fluxes (Table 2a, Fig. 4c). However, as suggested above for NH_4_^+^, such dark/light differences can also result from light induced fluctuations in the depth of the oxic/anoxic boundary.

Complementing nitrification in the silt that caused a release of NO_X_^−^, the estuarine sand removed NO_X_^−^ from the overlying seawater (Fig. 6a). The concentration gradient necessary for this influx was likely sustained by anaerobic metabolism: denitrification, and possibly anammox and DNRA. The latter would have led to retention of inorganic nitrogen^48–50^ but denitrification (and anammox) must have played a significant role because the NO_X_^−^ content of the seawater in both experimental units decreased over time (Table 1). The low BTSI (BTSI = 0) of the estuarine sand suggests a minor contribution by microalgae to sediment metabolism, leading to a relatively shallow and stable O_2_ penetration. These conditions support the anaerobic reduction of NO_X_^−^ via coupled nitrification–denitrification and denitrification of seawater NO_X_^−^. The relative contribution of these two pathways will have changed over the course of the experiment as the seawater NO_X_^−^ content decreased.

In conclusion, initially, the two sediments complemented each other in their inorganic nitrogen fluxes: the subtidal silt removed a small proportion of the NH_4_^+^ released by the estuarine sand, and the estuarine sand removed approximately the same amount of NO_x_^−^ as released by the subtidal silt. This initial coupling of estuarine sand and subtidal silt was not observed at the completion of the experiment because the direction and/or magnitude of sediment–seawater inorganic nitrogen fluxes had changed. We believe that the changes in flux that must have occurred in Control seawater simply follow the depletion of the initial seawater inorganic nitrogen content (Table 1). At the end of the experiment, the sediment’s pore water concentrations exceeded seawater concentrations, reversing the initial NO_X_^−^ uptake of the estuarine sand (Fig. 4a, Table 3c) and the initial NH_4_^+^ uptake of the subtidal silt (Figs 3c and 6b). Microalgae inhabiting both net heterotrophic sediments (BTSI = 1) may have intercepted upwards-diffusing NH_4_^+^ and NO_X_^−^ limiting the loss of inorganic nitrogen to the nitrogen depleted seawater (Fig. 6b). Furthermore, the initial supply of NH_4_^+^ from the overlying seawater to the silt’s diatoms had decreased over time, perhaps increasing the competition for upwards diffusing NH_4_^+^. If this inhibited nitrification, less NO_X_^−^ would have been generated further decreasing the NO_X_^−^ efflux. In the estuarine sand, the initial denitrification of seawater NO_X_^−^ had ceased as seawater [NO_X_^−^] decreased and nitrification of pore water NH_4_^+^ maintained the pore water [NO_X_^−^] above the seawater concentration causing efflux.

### Excess CO_2_ favours benthic microalgae and supresses AOB

Whereas the differences between the initial and final inorganic nitrogen fluxes in Control seawater can be explained by the observed changes in the Control seawater inorganic nitrogen content, those observed in Treatment seawater cannot. The increase in the silt’s BTSI and our O_2_ microprofiles suggest that the doubling of the pCO_2_ in Treatment seawater (Table 1) enhanced photosynthesis of benthic microalgae. In response to the low ambient pCO_2_ in the modern ocean, most marine microalgae, including diatoms, have evolved a carbon concentrating mechanism (CCM) to elevate concentrations at the site of carbon fixation^51–53^. An increase in pCO_2_ may cause down-regulation of microalgal CCM capacity^54,55^ and, given the energetic costs of this mechanism^56^, provide more energy for growth processes. The effect of excess CO_2_ on photosynthetic performance seems to be a function of the photosynthetic photon flux density (PPFD; excess CO_2_ under conditions of small PPFD may enhance algae growth while the opposite effect was observed under conditions of large PPFD^57–71^. In this context, it is important to acknowledge that while the LED lights in our experimental setup provided a PPFD that approximated the daytime PPFD measured at our subtidal site, this PPFD was an order of magnitude smaller than what algae in the estuarine sand can experience at the intertidal site.

The conditions in the Treatment seawater reversed the NO_X_^−^ efflux from the subtidal silt but left the NO_X_^−^ uptake of the estuarine sand unchanged (Fig. 6c). As described above the silt’s initial NO_X_^−^ efflux was likely driven by nitrification of NH_4_^+^ diffusing upwards from the anoxic silt and the pore water [NH_4_^+^] may have been highest at the oxic/anoxic boundary^46,72^, away from the photic zone. Initially, competition for NH_4_^+^ between pennate diatoms and AOB may have been minor (see above) and the diatom’s photosynthetic oxygenation of the pore water supported nitrification. The [NO_X_^−^] in the overlying seawater decreased over time, which should have increased the efflux of NO_X_^−^ from the silt. At completion of the experiment, however, the flux had reversed, apparently because the [NO_X_^−^] in the upper pore water had decreased below the concentration in the overlying seawater. We propose that two factors caused this decrease, the inhibition of nitrification combined with increasing NO_X_^−^ assimilation by microalgae. Both factors can lead to retention of nitrogen as they limit the availability of nitrate for denitrification. Microalgae can take up nitrogen faster and grow faster than AOB^30^ and so supress the growth and survival of AOB by nitrogen limitation in the oxic zone and O_2_ limitation in darkness, inhibiting coupled nitrification–denitrification^73–75^. The diatom photosynthesis caused considerable diel fluctuations in the oxygenation of the silt’s pore water (Fig. 5d). In darkness, O_2_ diffused from the seawater overlying the silt, across the diffusive boundary layer, and into the silt’s pore water to a depth of only about 4 mm (Table 3). Under conditions of light, however, photosynthetic O_2_ supersaturated the pore water in the upper silt, from where it diffused into the overlying seawater and deeper silt, oxygenating the pore water to a depth of about 9 mm. This has likely forced anaerobic denitrification activity deeper into the silt and decreased the diffusive supply of nitrate to denitrifiers from the silt-overlying seawater^30^.

The activity of benthic AOB is restricted to the upper few millimetres of oxygenated pore water^46,76^ and in our subtidal silt, AOB share this environment with motile pennate diatoms species. These and other diatom species can migrate deeper into the silt (for a review, see Consalvey and Paterson^77^) to exploit NH_4_^+^-rich pore water at the oxic/anoxic boundary thus limiting the supply of the NH_4_^+^ for AOB. This suspected nitrogen limitation of AOB is consistent with the results of analyses of field denitrification data from 18 European estuaries and laboratory studies, which revealed a general trend of lower coupled nitrification–denitrification rates in sediments populated by microalgae and inhibition of coupled nitrification–denitrification through nitrogen limitation^29^.

Besides possible NH_4_^+^ limitation of AOB and photosynthesis-induced inhibition of nitrification and denitrification, nitrification may have decreased following a decrease in the pH of the pore water at the oxic/anoxic boundary. The latter has still to be demonstrated, but if in fact the silt’s redox chemistry failed to buffer the pore water pH, the availability of uncharged ammonia, the substrate for the first step of nitrification^78,79^, may have limited nitrification (NH_3_ + H^+^ <−> NH_4_^+^; pKa = 9.3^80^). Evidence for such effect has been presented by others for the acidified seawater column^81–84^ and permeable sediment^7^. Furthermore, pore water acidification may affect the distribution of other reactive compounds such as iron and manganese, which could affect the cycling of nitrogen.

The suspected microalgae-induced inhibition of nitrification and denitrification, and enhanced assimilation of inorganic nitrogen by microalgae may have reduced the silt’s capacity to recycle dissolved nitrogen into the atmosphere^31,33,85–87^. Such an effect could link our evidence for CO_2_-enhanced benthic photosynthesis with the observed retention of inorganic nitrogen in the Treatment experimental unit. The magnitude and direction of the inorganic nitrogen fluxes measured initially and at the completion of the experiment, however, cannot readily explain why at the end of the experiment, Treatment seawater had retained more NO_x_^−^, (and NH_4_^+^) than Control seawater (Table 1). If the initial seawater inorganic nitrogen content was in equilibrium with the fluxes across the air– and sediment–seawater boundaries, then its depletion in the Control experimental unit must have been caused by a decrease in the release or an increase in uptake. Such a trend was not apparent and so it remains unclear what caused this depletion. Identifying this cause is further complicated by an unexpected confounding variable, the presence of juveniles of the heart urchin *Echinocardium cordatum* in 4 of 5 cores of *subtidal silt* in Control. The juveniles, which initially did not alter the smooth surface of the silt and therefore remained undetected, must have displaced some silt and mixed pore water^88^. Comparison of the *initial* silt–seawater fluxes of NH_4_^+^ or NO_X_^−^ in Control and Treatment did not reveal significant differences, indicating that at least initially their presence did not affect the cycling of inorganic nitrogen. Whether these so-called biodiffusors altered the microbial oxidation and reduction of nitrogen during the course of the experiment, we do not know. Such effects, however, are likely^11,21–26,89–92^.

In summary, our study, to the best of our knowledge, provides the first evidence for CO_2_-enhanced photosynthesis at the surface of a microalgae dominated, subtidal silt and reversal of the silt–seawater flux of NO_X_^−^, with potentially far-reaching consequences for benthic and pelagic productivity and underlying biogeochemical cycles. We hypothesise that in our experiment CO_2_-enhanced growth of benthic microalgae reduced the sediment’s capacity for coupled nitrification–denitrification. If this mechanism is correct, then CO_2_ enrichment of coastal seawater may decrease the recycling of N_2_ to the atmosphere adding to the pressures of already increasing coastal eutrophication.

## Material and Methods

### Sediment sampling

We collected ten cores of sediment from each of two sampling sites on the east coast of New Zealand’s North Island: subtidal silt on May 10^th^, 2015 at 12 m water depth in Man O’War Bay (S36° 47′38′′, E175° 10′14′′), Hauraki Gulf, and estuarine sand on May 12^th^, 2015 from a mid-intertidal sandflat (S37° 29′29′′, E175° 56′51′′) in the northern basin of Tauranga Harbour. These sediment types span the range of grain sizes typically found in New Zealand coastal habitats^36–38^. To collect a sediment core, we vertically pushed an acrylic tube (height = 30 cm, inner diameter = 9 cm) into the sediment until two-thirds of the tube was filled with sediment and then closed both ends with O-ring sealing lids. The sediment cores were stored on ice during their 3 h transport to the laboratory.

### Laboratory setup

The Control and Treatment experimental units each circulated ~560 L of synthetic seawater prepared with Coral Pro Salt (Red Sea, manufacturers specifications: salinity 35, pH 8.2–8.4, alkalinity 4.4–4.5 mEq L^−1^, calcium 0.455–0.475 g L^−1^, magnesium 1.36–1.42 g L^−1^, potassium 0.39–0.41 g L^−1^). Note that the alkalinity of this seawater exceeds that at our sampling sites, ~2.3 mEq L^−1 ^^41^. Each unit consisted of a pump submerged in a plastic incubation tank (112 × 72 × 60 cm), pumping ~9 L min^−1^ through a water cooler and UV sterilizer into an elevated 210 L mixing barrel from which the seawater returned to the incubation tank by gravity (Fig. 1). The cooler kept the seawater temperature, measured continuously by a platinum resistance thermometer, at 19.7 ± 0.3 °C (Mean ± 1 SD, n = 7162). Seawater was also circulated between a particle filter and the incubation tank. Jets from the mixing barrel and the particle filter caused sufficient turbulence to fully mix the seawater overlying the sediment. One LED floodlight per experimental unit provided photosynthetically active radiation to the surface of the sediment cores from 7 am to 7 pm. The photosynthetic photon flux density (PPFD) at the sediment surface, measured with a planar underwater quantum sensor flush with the sediment surface, was like that measured midday at our subtidal site, ∼130 μmol quanta m^−2^ s^−1^. The PPFD at the sediment surface of our intertidal site can be an order of magnitude higher.

### Seawater carbonate system

A SenTix HWD electrode connected to a pH 3310 meter (WTW) continuously measured and transmitted the pH of the seawater inside the mixing barrel to a laptop running software, which opened and closed a solenoid valve if the pH increased above or decreased below the setpoint of pH 8.1 (Control) or 7.9 (Treatment, Fig. 1). The solenoid valve controlled a stream of CO_2_-enriched air (5% carbon dioxide, 21% oxygen in nitrogen) from a 9 m^3^ gas cylinder to an air stone at the bottom of the mixing barrel. A second identical set of pH meter/electrode continuously measured and transmitted the pH of the seawater surrounding the sediment cores in the incubation tank.

We used three-point calibrations with NIST/DIN pH buffers to test for theoretical Nernstian electrode behaviour, then conditioned the electrodes in seawater before determining the electrode-specific offset between the potential measured in NIST/DIN pH buffer and in certified seawater reference material (TRIS in synthetic seawater, Batch #26), which we obtained from A. Dickson, Marine Physical Laboratory, Scripps Institution of Oceanography^93^. The electrodes were cleaned, refilled and recalibrated three times during the experiment.

To determine other seawater carbonate system parameters, we collected a single 1 L water sample from each unit twice a week during the first four weeks and once a week for the remaining three weeks of the experiment and measured the concentration of dissolved inorganic carbon following Dickson *et al*.^93^. The seawater total alkalinity and pCO_2_ were derived with the USGS CO_2_calc Application^94^ (CO_2_ constants = K1, K2^95^; pH scale = total scale, mol kg SW^−1^). We measured the seawater salinity with a conductivity meter and maintained it within the range 35 ± 0.3 (Mean ± 1 SD, n = 40) by adding distilled water.

Injection of CO_2_-enriched air initially stabilized the seawater pH in Control and Treatment at 8.1. The following stepwise increase of this injection in the Treatment increased the seawater pCO_2_ and dissolved inorganic carbon concentration by a factor of 2.4 and 1.06, respectively, and decreased the seawater pH to 7.87 (Table 1). The pH setpoint for the mixing barrel of the Treatment was 7.8 but the departure from seawater–air equilibrium caused CO_2_ degassing, which offset the pH of the seawater in the incubation tank by 0.07 pH units.

### Sediment–seawater nitrogen and O_2_ flux and pore water oxygenation

Measurements of sediment–seawater solute fluxes were made at the beginning (*initial*) and at the end (*final*) of the experiment (Fig. 1) during the last 4 h of a light period and then the last 4 h of the following dark period. To quantify the fluxes of NH_4_^+^, and NO_X_^−^, we removed 100 mL aliquots of the seawater overlying the sediment in each acrylic tube before and after a 4-h incubation, during which we isolated the sediment-overlying seawater from the surrounding tank seawater with O-ring sealing lids. The aliquots were immediately filtered (0.45 µm pore size) and then kept frozen until analysed with standard methods for seawater^96^ using an air-segmented continuous flow auto-analyser. We also included acrylic tubes that contained seawater but not sediment to correct for water column processes. The before-and-after difference in [NH_4_^+^] and [NO_X_^−^], the volume of the enclosed seawater, and the size of the visible sediment surface area were then used to derive the sediment–seawater solute flux (μmol m^−2^ h^−1^).

During the 4-hour incubations, a peristaltic pump circulated the seawater enclosed in each core through Teflon tubes connected to one inlet and one outlet port in the sealing lid. The purpose of this circulation was to avoid stagnation of the enclosed sediment-overlying seawater and to measure changes in its oxygenation with a miniaturized in-line chemical optical sensor connected to a Bluetooth transmitter, which transferred continuous sensor readings to a laptop. The total O_2_ uptake (TOU, µmol m^−2^ h^−1^) between sediment and seawater was calculated from the slope of the [O_2_] time-series (r^2^ > 0.98), the volume of the enclosed seawater and the area of the visible sediment surface. TOU included the effects of benthic fauna and microalgae on O_2_ exchange: mixing of sediment pore water with bottom seawater due to displacement of sediment particles, ventilation of faunal burrows, photosynthesis and respiration. To assess the oxygenation of the silt’s pore water, we measured 10 vertical O_2_ concentration microprofiles in each of two cores, one submerged in Control seawater and one in Treatment seawater, at the beginning and the end of the experiment. These microprofiles were measured with Unisense A/S hard- and software under conditions of light and darkness at 0.1 mm resolution starting in the sediment-overlying seawater to the depth of the anoxic sediment. Because shell fragments in the estuarine sand broke the fragile microsensors, profiling measurements could not be performed in the estuarine sand.

### Sediment analyses

After the final solute flux measurements, we gravity siphoned off the overlying seawater in five Control and five Treatment cores of each sediment type and then removed and homogenized the surface 2 cm of sediment. To analyse the particle size distribution (Malvern Mastersizer 2000 particle analyser), we removed ~9 mL from the homogenized samples of three haphazardly chosen Control and three Treatment cores per sediment type. To determine the sediment water and organic matter content, we combined the remaining samples to make one Control and one Treatment sample for each sediment type and analysed each of these four samples in quintuplicates after drying at 90–100 °C for 24 h, and after combustion at 400 °C in a muffle furnace for 6 h.

### Data analyses

To characterize the overall sediment metabolism, we used the benthic trophic state index (BTSI^40^), which classifies sediment into four different categories and reflects a gradient of increasing algal activity: BTSI = 0 (fully heterotrophic), no statistically significant difference between TOU_light_ and TOU_dark_ and a numerical difference ≤ 25%; BTSI = 1 (net heterotrophic), 0 < TOU_light_ < TOU_dark_; BTSI = 2 (net autotrophic), TOU_light_ < 0 and |TOU_light_| < TOU_dark_; and BTSI = 3 (highly autotrophic), TOU_light_ < 0 and |TOU_light_| > TOU_dark_.

The statistical analysis of our experiment is complicated by the repeated sampling of incubated cores and the four different conditions (factors) under which measurements were made; sediment type (estuarine sand, subtidal silt), pH (Control, Treatment), light regime (light, dark) and time (initial, final). Preliminary analyses that included all factors indicated significant higher (third and fourth) order interaction terms for all response variables (TOU, ∆TOU, and NH_4_^+^ and NO_X_^−^ fluxes), which complicates interpretation. Accordingly, we simplified our analysis whereby for each sediment type we conducted a series of repeated measures two-way analyses testing the interactive effects of two factors on a response variable, within the different levels of the third factor. For example, for each sediment type the interactive effects of pH and time was tested separately for the light and dark measurements. This approach allowed us to focus on the effects of pH, time and light regime on response variables, and to better illustrate response differences between sediment types.

Statistical analyses were undertaken with a two-way repeated measures permutational multivariate analysis of variance (PERMANOVA) using Euclidean distance matrices. The factors pH, time and light regime (all 2 levels) were treated as fixed factors and core (5 levels) as a random factor nested within pH treatment or light regime depending on the fixed factor combination tested. Main effects were not considered if the interaction term was significant (α < 0.05), instead post-hoc pair-wise tests were undertaken to identify differences between treatment effects for each level of the other factor. Analyses were performed using the PRIMER (with the PERMANOVA A + addition) statistical software program^37,97^. A one way PERMANOVA was used to test the significance of differences between the inorganic nitrogen content of the seawater circulating in the Treatment and Control experimental units.

### Data availability

The datasets generated during the current study are available from the corresponding author on reasonable request.

## Electronic supplementary material

Supplementary material
