# Supplementary material for: Effects of CO2 enrichment on benthic primary production and inorganic nitrogen fluxes in two coastal sediments
Source: Sci Rep. 2018 Jan 18;8:1035. doi: 10.1038/s41598-017-19051-w (PMC5773597; doi:10.1038/s41598-017-19051-w)
Supplement: Supplementary file 1 — Supplementary material [file 41598_2017_19051_MOESM1_ESM.pdf]

Supplementary material

Effects of CO_2 enrichment on benthic primary production and inorganic nitrogen fluxes in two coastal sediments

Kay Vopel, Cintya Del-Río and Conrad A. Pilditch

Table S1. Sediment properties. Average (± 1 s.d.) granulometric indices (n = 3) and water and organic matter contents (n = 5) of poorly sorted estuarine sand and subtidal silt cores submerged in Control and Treatment seawater.

	Estuarine sand		Subtidal silt	
	Control	Treatment	Control	Treatment
Median grain size (µm)	323 ± 13	332 ± 46	31 ± 1	29 ± 2
Lower Quartile (µm)	513 ± 40	525 ± 43	61 ± 6	56 ± 6
Upper Quartile (µm)	208 ± 16	213 ± 8	9 ± 0	9 ± 0
Inclusive Sorting Coefficient	1.17 ± 0.03	1.24 ± 0.08	1.98 ± 0.07	1.90 ± 0.08
Inclusive Graphic Skewness	0.21 ± 0.03	0.20 ± 0.06	0.08 ± 0.02	0.05 ± 0.03
Silt/clay content (% volume <63 µm)	7.75 ± 0.95	8.29 ± 1.87	79.43 ± 3.23	79.05 ± 1.31
Water content (% wet weight)	24.6 ± 1.0	24.4 ± 1.0	73.7 ± 0.2	74.1 ± 0.3
Organic matter content (% dry weight)	1.2 ± 0.2	1.3 ± 0.1	5.6 ± 0.3	6.4 ± 0.3

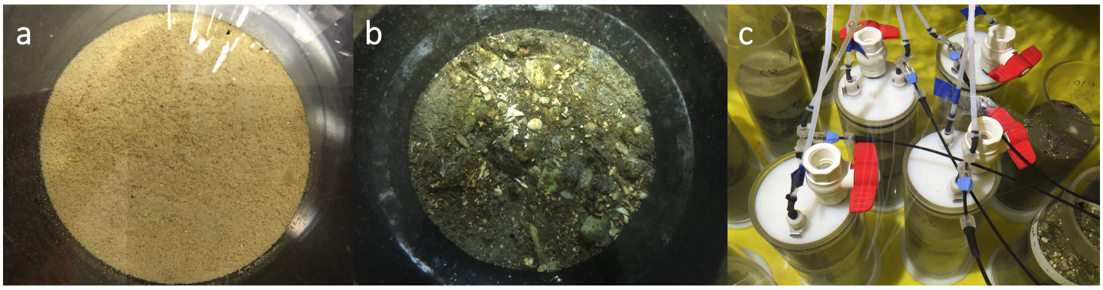

Figure S1. Photographs showing the surfaces of a subtidal silt (a) and a estuarine sand core (b), and four cores sealed for solute flux measurements (c). The surface of the estuarine sand featured numerous shell fragments, small extruding tubes and exposed cockle shells. The estuarine sand contained 1–3 small clams (*Austrovenus stutchburyi*) in 8 of the 10 cores, and 1–2 larger polychaetes in 6 of the cores. One Ambient core contained 7 clams and another did not contain any large macrofauna. Motile benthic diatom species of the genera *Pleurosigma*, *Gyrosigma, Nitzschia, Thalassionema*, *Bacillaria* and others populated the surface of the subtidal silt cores, which initially was smooth with no obvious macrofaunal trails, tubes or burrow openings. The subtidal silt contained one small gastropod snail in one Treatment core, two small bivalves in another Treatment core, and one juvenile of the irregular sea urchin *Echinocardium cordatum* (1.2–1.7 cm diameter) in each of three Ambient cores. Two larger individuals of *E. cordatum* (2.5 and 3.5 cm diameter) occurred in one Ambient core.
